# Synovial inflammation and immune-stromal crosstalk in osteoarthritis-related synovial fibroblast research, 2006–2025: a bibliometric analysis

**DOI:** 10.3389/fimmu.2026.1898740

**Published:** 2026-10-01

**Authors:** Tiange Xia, Xiaoyan Sun, Zhiwei Jiang, Yijie Zhang, Yi Zhou, Yang Shao, Shaoshuo Li, Jianwei Wang

**Affiliations:** 1 Nanjing University of Chinese Medicine, Nanjing, China; 2 Wuxi TCM Hospital Affiliated to Nanjing University of Chinese Medicine, Wuxi, China

**Keywords:** bibliometric analysis, fibroblast-like synoviocytes, immune-stromal crosstalk, osteoarthritis, science mapping, single-cell analysis, synovial fibroblasts, synovitis

## Abstract

**Background:**

Osteoarthritis (OA) is increasingly understood as a whole-joint organ disease involving cartilage, synovium, subchondral bone, and the local immune-stromal microenvironment. Synovial fibroblasts and fibroblast-like synoviocytes (FLS) participate in synovitis, matrix remodeling, cartilage-synovium crosstalk, pain-associated microenvironments, and disease heterogeneity. However, the knowledge structure, main knowledge sources, and frontier evolution of OA-related synovial fibroblast research remain incompletely mapped.

**Methods:**

We retrieved publications on OA-related synovial fibroblasts/fibroblast-like synoviocytes (OA-FLS) published from 2006 to 2025 from the Web of Science Core Collection (WOSCC) and Scopus. After integration and deduplication, Bibliometrix, CiteSpace, VOSviewer, and Python were used to analyze annual publication trends, national and institutional contributions, author and journal distributions, collaboration networks, global and local citation structures, keyword co-occurrence, trend topics, and thematic evolution.

**Results:**

A total of 1,881 publications were included, comprising 1,642 articles and 239 reviews. Annual output increased from 53 publications in 2006 to 142 in 2025, with an annual growth rate of 5.32%. China contributed the largest number of publications, whereas international collaboration remained relatively limited. Keyword and citation structures indicated a strong influence of RA-FLS-related knowledge sources. Recent hotspots increasingly involved synovitis, immune-stromal interaction, extracellular vesicle-mediated communication, fibrosis, pain-associated fibroblast states, and single-cell/spatially related studies.

**Conclusion:**

OA-FLS research has expanded from early studies of inflammatory mediators and catabolic pathways toward questions about fibroblast states, tissue location, immune-stromal interaction, and clinical phenotype association. These findings suggest a gradual movement from RA-derived conceptual borrowing toward more OA-contextualized interpretation, although this pattern should be understood as a bibliometric inference rather than direct mechanistic proof. Future studies need to clarify which OA-FLS states have stable, reproducible, and pathologically meaningful disease associations through standardized sampling, multicenter validation, spatial localization, functional experiments, and integration with clinical phenotypes.

## Introduction

Osteoarthritis (OA) is one of the most common chronic joint diseases and is mainly characterized by joint pain, functional limitation, and structural damage (1, 2). Traditionally, OA was regarded as a degenerative disease centered on cartilage degeneration and “wear and tear,” but this framework does not adequately explain its clinical heterogeneity: pain, synovitis, imaging-defined structural damage, and treatment response often do not show a linear correspondence (1, 3, 4). In recent years, OA has increasingly been redefined as a whole-joint organ disease involving the synovium, cartilage, subchondral bone, and periarticular tissues. Mechanical, inflammatory, and immune-stromal interactions among these tissues jointly contribute to disease onset and progression (1, 3, 4).

Within this conceptual shift, the importance of the synovium and its cellular composition has been reassessed. Synovitis can be observed at different stages of OA and is associated with pain, joint swelling, and progression of cartilage damage (5, 6). Synovial fibroblasts in the synovial lining and sublining layers contribute to tissue homeostasis and can respond to inflammatory mediators, mechanical stimulation, metabolic abnormalities, and damage-associated signals, thereby regulating cytokine and chemokine secretion, matrix degradation and remodeling, cartilage-synovium crosstalk, and fibrotic changes (6–8). In this article, OA-related synovial fibroblasts and fibroblast-like synoviocytes are collectively referred to as OA-FLS. After this definition, OA-FLS is used as the collective term for OA-related synovial fibroblasts/fibroblast-like synoviocytes, whereas FLS is used as the abbreviation for fibroblast-like synoviocytes where appropriate. Existing studies suggest that OA-FLS are not merely structural support cells but active cell populations involved in persistent synovitis, pain-associated states, local microenvironmental shaping, and tissue remodeling (7–9).

However, OA-FLS research did not develop within a fully independent knowledge system. The rheumatoid arthritis (RA) field developed a relatively mature RA-FLS research framework earlier, covering fibroblast activation, migration and invasion-like phenotypes, perpetuation of inflammation, Toll-like receptor-related inflammatory signaling, non-coding RNA regulation, and functional subset definition (10–14). These studies have provided conceptual resources and methodological references for OA-FLS research, but they also raise a question that needs clarification: to what extent has OA-FLS research borrowed the conceptual language and technical paths of RA-FLS research, and where has it gradually formed an interpretation aligned with the pathological features of OA itself (7, 13, 14)?

This issue is not simply a matter of terminology. The core pathology of RA is autoimmune chronic erosive synovitis, whereas OA more often occurs in the context of aging, mechanical loading, metabolic abnormalities, joint injury, low-grade inflammation, and chronic tissue remodeling (4, 15). Therefore, even when the two diseases share certain molecular pathways, cellular markers, or phenotypic terms, they may differ substantially in triggering conditions, tissue localization, and clinical meaning (9, 13, 14, 16). OA-FLS should be understood within the tissue ecology and clinical heterogeneity of OA itself, rather than being treated as a direct extension of the RA-FLS framework.

In recent years, OA-FLS-related studies have continued to increase. The field has expanded from traditional synovitis and inflammatory mediators to non-coding RNA, epigenetic regulation, extracellular vesicle-mediated cartilage-synovium crosstalk, metabolic and mechanical signaling, fibrosis, and single-cell-level heterogeneity analysis (7–9, 11, 17, 18). Narrative reviews can summarize selected mechanistic advances, but they are less able to systematically delineate the macro-level knowledge structure of the field, including the major contributing countries, institutions, authors, and journals; the knowledge base and disciplinary sources; the research shifts reflected by keyword and citation networks; and whether the collaboration landscape can support comparisons and validation across cohorts, platforms, and disease stages (19–21). Although bibliometric approaches have been applied to broader OA synovium-related topics (22), OA-FLS research has not, to our knowledge, been specifically mapped as a distinct knowledge domain. Therefore, the present study differs by focusing on OA-FLS as the core search and interpretation unit, integrating WOSCC and Scopus records, and examining how this field has been shaped by RA-FLS-derived knowledge while developing more OA-contextualized research themes.

Bibliometric analysis offers a suitable broad perspective for addressing these questions because it can simultaneously examine publication output, collaboration patterns, citation structures, and thematic evolution (19–21). Accordingly, this study integrated OA-FLS-related publications from 2006 to 2025 in the WOSCC and Scopus databases and systematically analyzed global output trends, collaboration networks, core authors and journals, citation structures, keyword hotspots, and thematic evolution. We aimed to clarify how OA-FLS research has developed, where its main knowledge sources lie, and which directions may require more standardized samples, cross-cohort validation, and clinical phenotype linkage.

## Materials and methods

### Data sources and data processing

Bibliographic records included in this study were retrieved from the Web of Science Core Collection (WOSCC) and Scopus databases to enhance the comprehensiveness and robustness of the analysis. The retrieval period covered publications from January 1, 2006, to December 31, 2025. Only English-language publications were included, and document types were restricted to Articles and Reviews. All searches and data exports were completed on May 2, 2026, to reduce record discrepancies caused by ongoing database updates.

The search strategy was constructed using Boolean logic to capture studies related to osteoarthritis and synovial fibroblasts as comprehensively as possible. The specific search formulas were as follows:

TS=((“osteoarthrit*” OR “osteoarthros*” OR “degenerative joint disease*” OR “degenerative arthritis”) AND (“synovial fibroblast*” OR “fibroblast-like synoviocyte*” OR “fibroblast like synoviocyte*” OR “fibroblast-like synovial cell*” OR “fibroblast like synovial cell*” OR “type B synoviocyte*” OR “type-B synoviocyte*” OR “osteoarthritis synovial fibroblast*” OR “osteoarthritic synovial fibroblast*” OR “osteoarthritis fibroblast-like synoviocyte*” OR “osteoarthritic fibroblast-like synoviocyte*”))

and:

TITLE-ABS-KEY((“osteoarthrit*” OR “osteoarthros*” OR “degenerative joint disease*” OR “degenerative arthritis”)) AND TITLE-ABS-KEY((“synovial fibroblast*” OR “fibroblast-like synoviocyte*” OR “fibroblast like synoviocyte*” OR “fibroblast-like synovial cell*” OR “fibroblast like synovial cell*” OR “type B synoviocyte*” OR “type-B synoviocyte*” OR “osteoarthritis synovial fibroblast*” OR “osteoarthritic synovial fibroblast*” OR “osteoarthritis fibroblast-like synoviocyte*” OR “osteoarthritic fibroblast-like synoviocyte*”)) (**Supplementary Table S1**).

The search strategy was intentionally centered on synovial fibroblasts and FLS to preserve thematic specificity. Broader terms such as “stromal cells,” “mesenchymal stromal cells,” and “synovial lining cells” were not included as independent search terms because they may retrieve heterogeneous stromal, mesenchymal, or lining-cell studies that do not specifically address OA-FLS. This choice improved conceptual specificity but may have reduced coverage of studies indexed under broader stromal-cell terminology.

The searches initially identified 2,026 WOSCC and 1,809 Scopus records; after eligibility screening, 1,592 and 1,339 records, respectively, remained for integration and deduplication. Data integration and deduplication followed standardized workflows widely adopted in previous bibliometric studies (23, 24). Specifically, the Bibliometrix package and custom Python scripts were used to merge datasets and remove duplicate records. Duplicate records were identified through a stepwise procedure. Records with identical DOI or identical normalized titles were first identified automatically. For records without DOI, potential duplicates were then screened by normalized title, first-author name, publication year, and journal information, followed by manual confirmation. Title normalization included conversion to lowercase, removal of punctuation, and trimming of extra spaces. Potential duplicates with inconsistent metadata were inspected manually, with priority given to DOI, complete title, first-author information, journal, and publication-year consistency. PMID was not used as a mandatory matching field because it was not consistently available across WOSCC and Scopus exports. Ultimately, 1,881 unique publications were included in the final analysis, including 1,642 articles and 239 reviews (**Figure 1**). The complete search strategies for WOSCC and Scopus are provided in **Supplementary Table S1**. Titles, abstracts, and keywords of all included publications were integrated into a unified corpus for subsequent analyses.

**Figure 1:**
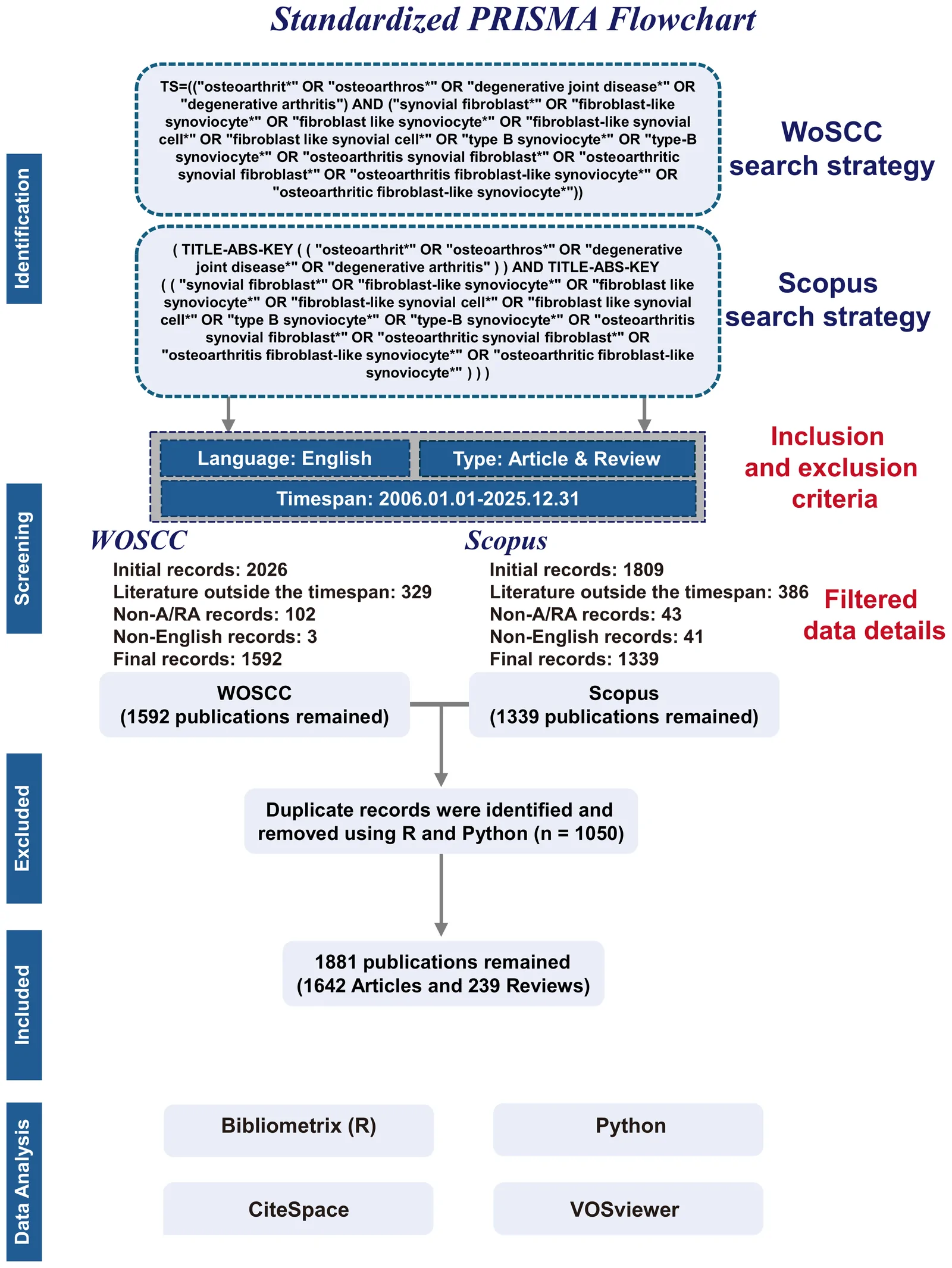
Literature identification, database integration, and deduplication workflow. The image summarizes retrieval, integration, deduplication, and final inclusion of records from WOSCC and Scopus.

Before formal analysis, author, institution, country/region, journal, and keyword fields were standardized. Standardization was performed using a combination of automatic cleaning and manual inspection. For author names, obvious spelling variants, initials, capitalization differences, and formatting inconsistencies were checked and harmonized where the identity was clear. For institutions, common name variants and known changes in institutional naming were merged when they clearly referred to the same entity; otherwise, the original database record was retained to avoid overcorrection. Country and region names were standardized according to the exported affiliation fields and manually checked for common variants. Keywords were harmonized by merging obvious singular/plural forms, abbreviation variants, and synonymous expressions where appropriate. Because complete author disambiguation is not possible in bibliometric datasets, potential homonyms and author-name ambiguity were acknowledged as methodological limitations.

### Bibliometric analysis

A multidimensional bibliometric framework was used to systematically characterize the knowledge structure and developmental trajectory of this field. Descriptive bibliometric analyses, including annual publication trends, citation patterns, leading countries, prolific authors, journal distributions, and citation analyses, were performed using the Bibliometrix package (v5.0) in R software (v4.4.5). Because WOSCC and Scopus differ in citation coverage, indexing policies, and metadata structures, citation-related indicators were interpreted descriptively rather than as strictly interchangeable measures. Database-specific descriptive indicators were first summarized separately and then compared with the merged dataset. In the merged analysis, global citation counts, local citation counts, and normalized citation indicators were calculated within the integrated corpus when available. We did not attempt to force equivalence between WOSCC and Scopus citation counts; instead, citation analyses were used to identify broad knowledge sources and internal citation structures.

Trend topic analysis was conducted using the Trend Topics function in Bibliometrix. The top 120 high-frequency terms were extracted, and thematic clustering was performed using the Louvain algorithm with a minimum frequency threshold of 5 per thousand publications. Three representative labels were assigned to each thematic cluster.

Author and country collaboration networks were also constructed using Bibliometrix, with Louvain clustering, association-strength normalization, and automatic layout optimization. Visualization parameters were set as follows: maximum number of nodes = 50, repulsion parameter = 0.5, minimum edge weight = 1, exclusion of isolated nodes, and retention of authors with more than 20 publications.

Keyword clustering analysis was further performed using CiteSpace (v6.2.R1). The time slicing interval was set to one year, and node selection parameters were as follows: g-index (k = 25), LRF = 2.5, L/N = 10, LBY = 5, and e = 1.0. The Minimum Duration for keyword burst and citation burst analyses was set to 2, while the remaining algorithmic parameters, including the state transition rate alpha and weight gamma, were kept at their default settings (alpha = 2.0, gamma = 1.0) (25). In addition, VOSviewer (v1.6.20) was used to construct author co-citation and journal co-citation networks. The threshold criteria were author co-citation frequency at least 100 and journal co-citation frequency at least 500 (26). Selected raw outputs generated by the above software were subsequently imported into a Python environment for advanced visualization and network rendering using the pandas, matplotlib, seaborn, and networkx libraries.

## Results

### Overall bibliometric characteristics and annual growth trends

Between 2006 and 2025, 1,881 OA-FLS-related publications were included after merging WOSCC and Scopus. The WOSCC dataset contained 1,592 publications, and the Scopus dataset contained 1,339 publications; after cross-database deduplication, the final corpus included 500 sources, 92,925 references, 10,164 authors, 2,961 author keywords, and 6,856 Keywords Plus terms. Keywords Plus refers to index terms generated from the titles of cited references. By document type, there were 1,642 articles and 239 reviews; the average number of citations per document was 43.17, and the percentage of international co-authorships was 17.49% (**Figures 1**, **2**; **Table 1**).

**Figure 2:**
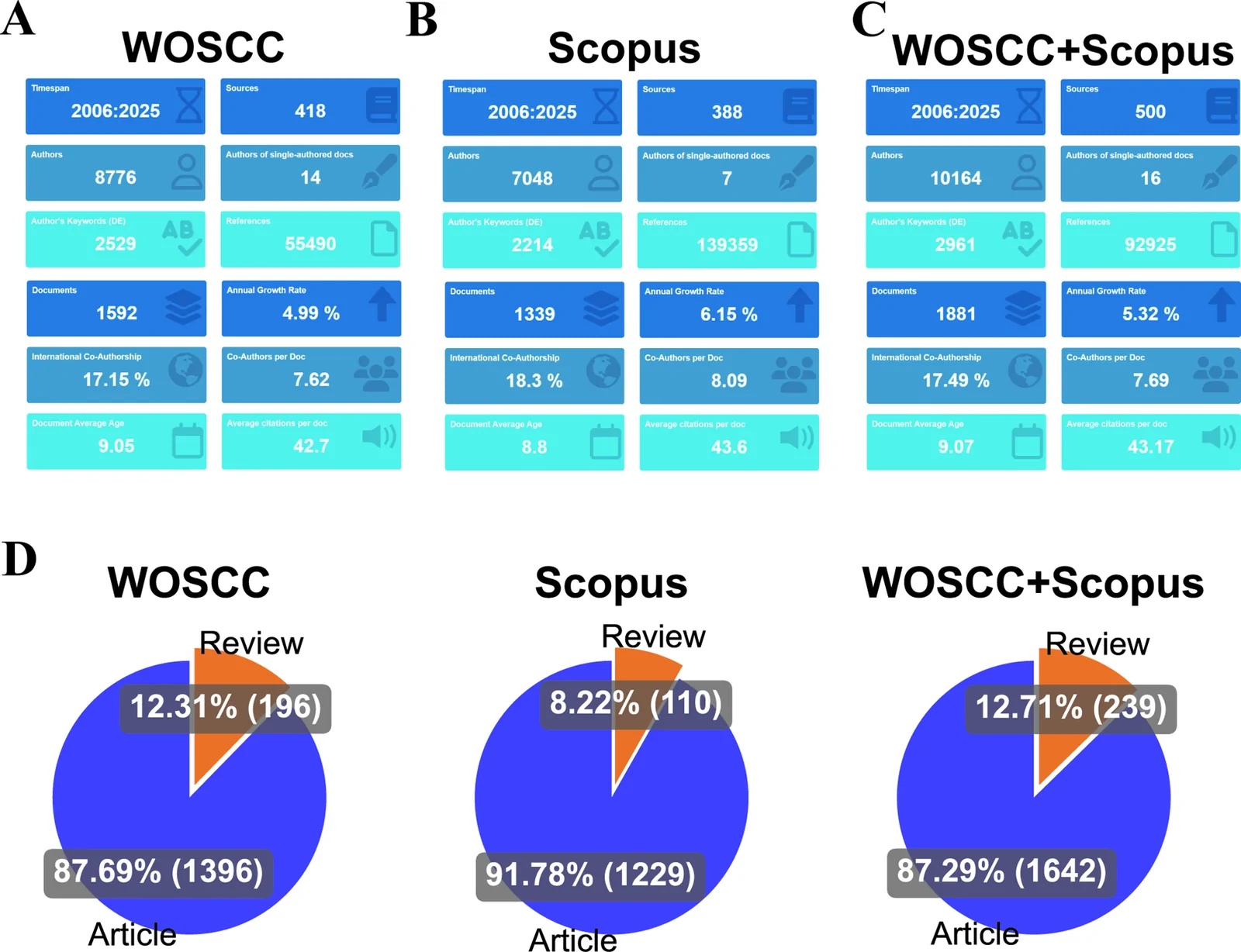
Overall bibliometric characteristics and document-type composition of WOSCC, Scopus, and the merged dataset. **(A–C)** Show the timespan, sources, authors, references, annual growth rate, international co-authorship percentage, and other indicators for the three datasets; the merged WOSCC+Scopus column represents 1,881 records after cross-database deduplication. **(D)** Shows the proportions of articles and reviews.

**Table 1:** Overall bibliometric characteristics of the WOSCC, Scopus, and merged datasets.

Description	WOSCC	Scopus	WOSCC+Scopus
Timespan	2006:2025	2006:2025	2006:2025
Sources (Journals, Books, etc.)	418	388	500
Documents	1592	1339	1881
Annual Growth Rate %	4.99	6.15	5.32
Document Average Age	9.05	8.8	9.07
Average citations per doc	42.7	43.6	43.17
References	55490	139359	92925
Keywords Plus (ID)	3409	9205	6856
Author’s Keywords (DE)	2529	2214	2961
Authors	8776	7048	10164
Authors of single-authored docs	14	7	16
Single-authored docs	17	7	20
Co-Authors per Doc	7.62	8.09	7.69
International co-authorships %	17.15	18.3	17.49
article	1396	1229	1642
review	196	110	239

Keywords plus refers to index terms generated from the titles of cited references.

Annual publication output showed an overall upward trend. The number of publications increased from 53 in 2006 to 142 in 2025, with an annual growth rate of 5.32%; after 2019, most years had more than 100 publications. Articles consistently accounted for the majority of publications, with 121 articles and 21 reviews in 2025. Average citations per document were higher in early years, reaching 89.55 in 2006, 71.03 in 2013, and 4.04 in 2025. Because the annual time series contained only 20 observations, forecasting analyses were treated as descriptive and exploratory rather than inferential. In multi-model comparisons, the third-order polynomial model had an RMSE of 8.40, lower than the second-order polynomial, linear, LSTM, and ARIMA models. Exploratory extrapolation based on historical data suggested that publication output may continue to increase (**Figure 3**). This extrapolation was used only to describe a possible continuation of previous growth patterns and should not be interpreted as a deterministic prediction of future publication output.

**Figure 3:**
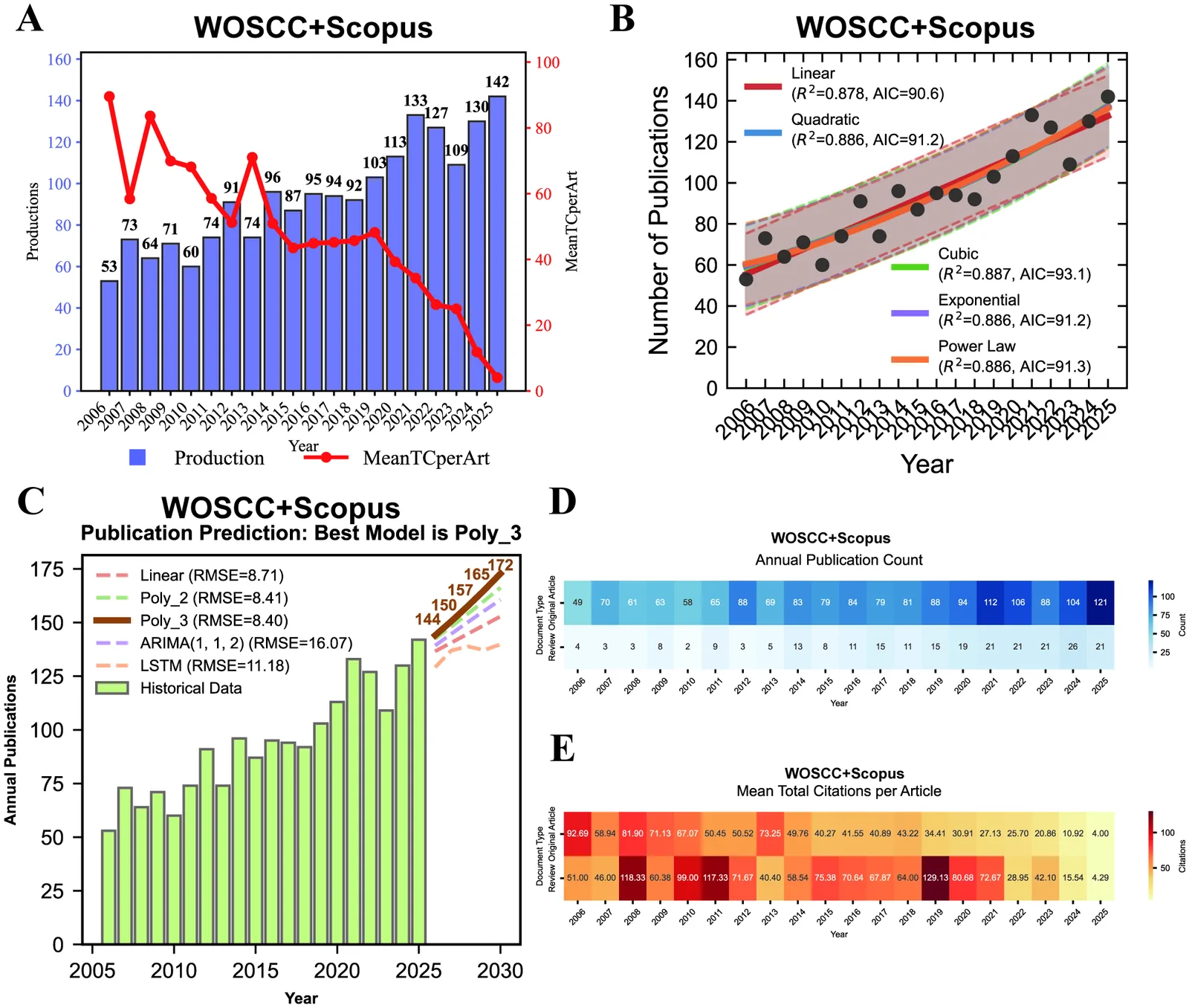
Annual publication trends, citation changes, and exploratory publication forecasting in OA-FLS research from 2006 to 2025. **(A)** Shows annual publication output and average citations per document; **(B, C)** show model fitting and exploratory extrapolation across different models; **(D, E)** show heatmaps of annual publication output and average total citations.

### Country, institution, author, and journal output patterns

At the country and regional level, China, the United States, Japan, Korea, and Germany were the main contributors to this field. China ranked first with 676 publications, accounting for 35.9% of all publications; the United States contributed 202 publications, Japan 187, Korea 123, and Germany 103 (**Table 2**). Publication scale, collaboration proportion, and citation impact differed across countries. China had a multiple-country publication percentage (MCP%) of 5.6%, lower than that of the United Kingdom, Switzerland, Germany, and Italy (**Figures 4B, C**; **Table 2**). MCP indicates multiple-country publications, whereas SCP indicates single-country publications.

**Table 2:** Output and international collaboration of leading countries/regions.

Country	Articles	Articles %	SCP	MCP	MCP %
CHINA	676	35.9	638	38	5.6
USA	202	10.7	156	46	22.8
JAPAN	187	9.9	169	18	9.6
KOREA	123	6.5	109	14	11.4
GERMANY	103	5.5	75	28	27.2
ITALY	68	3.6	49	19	27.9
UNITED KINGDOM	63	3.3	37	26	41.3
SWITZERLAND	57	3	28	29	50.9
SPAIN	46	2.4	36	10	21.7
FRANCE	45	2.4	34	11	24.4

SCP, single-country publications; MCP, multiple-country publications.

**Figure 4:**
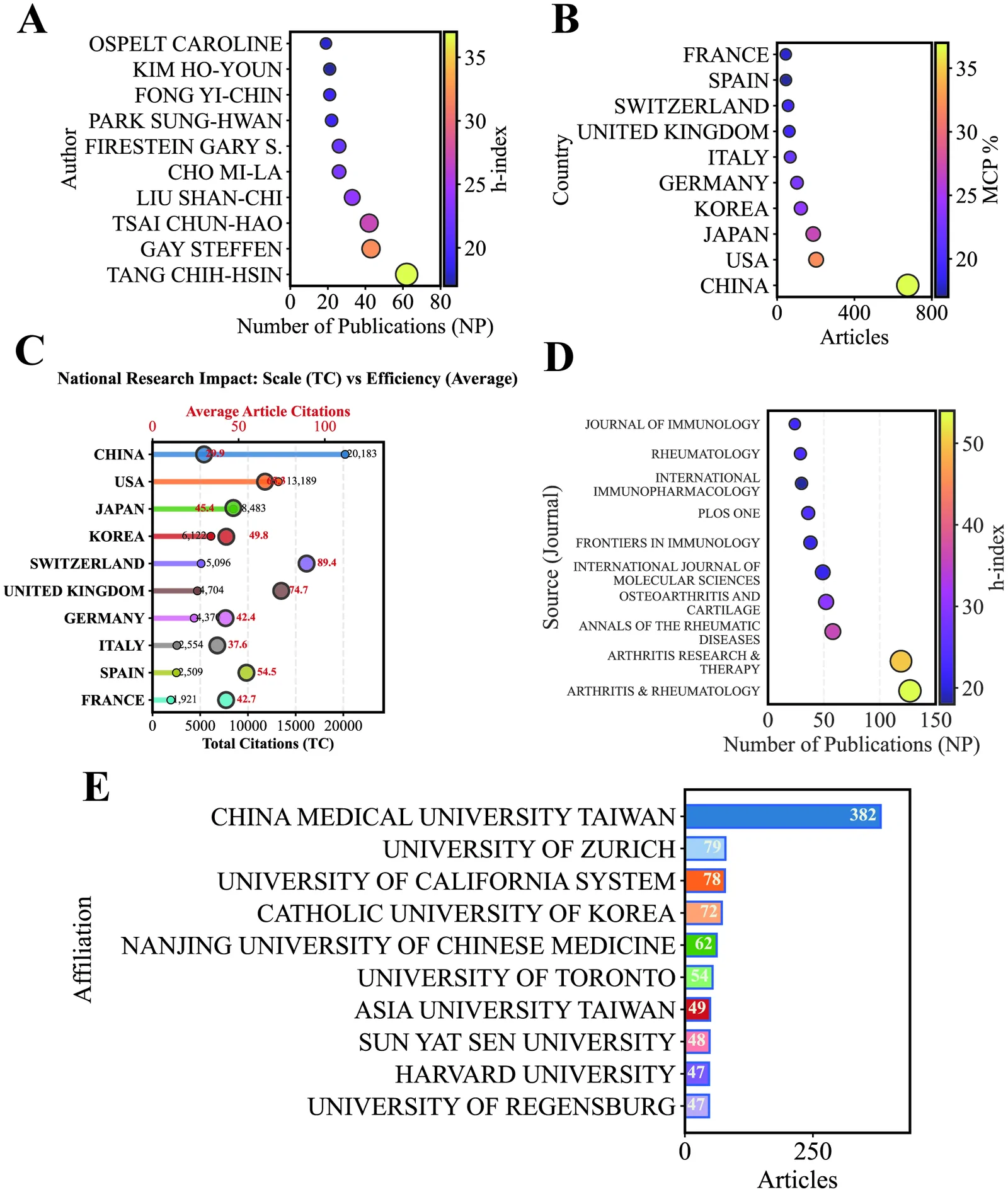
Contributions of leading authors, countries/regions, journals, and institutions. **(A)** Shows prolific authors and h-index; **(B)** shows leading countries/regions and MCP%; **(C)** shows total citations, average citations per document, and SCP/MCP composition by country/region; **(D)** shows leading journals by publication output and h-index; **(E)** shows publication output of leading institutions. MCP indicates multiple-country publications, and SCP indicates single-country publications.

At the institutional level, China Medical University Taiwan, University of Zurich, University of California System, Catholic University of Korea, Nanjing University of Chinese Medicine, University of Toronto, Asia University Taiwan, and Sun Yat Sen University ranked among the leading institutions. Institutions differed in publication volume, continuity of output, and positions within collaboration networks (**Figures 4E**, **5D**).

**Figure 5:**
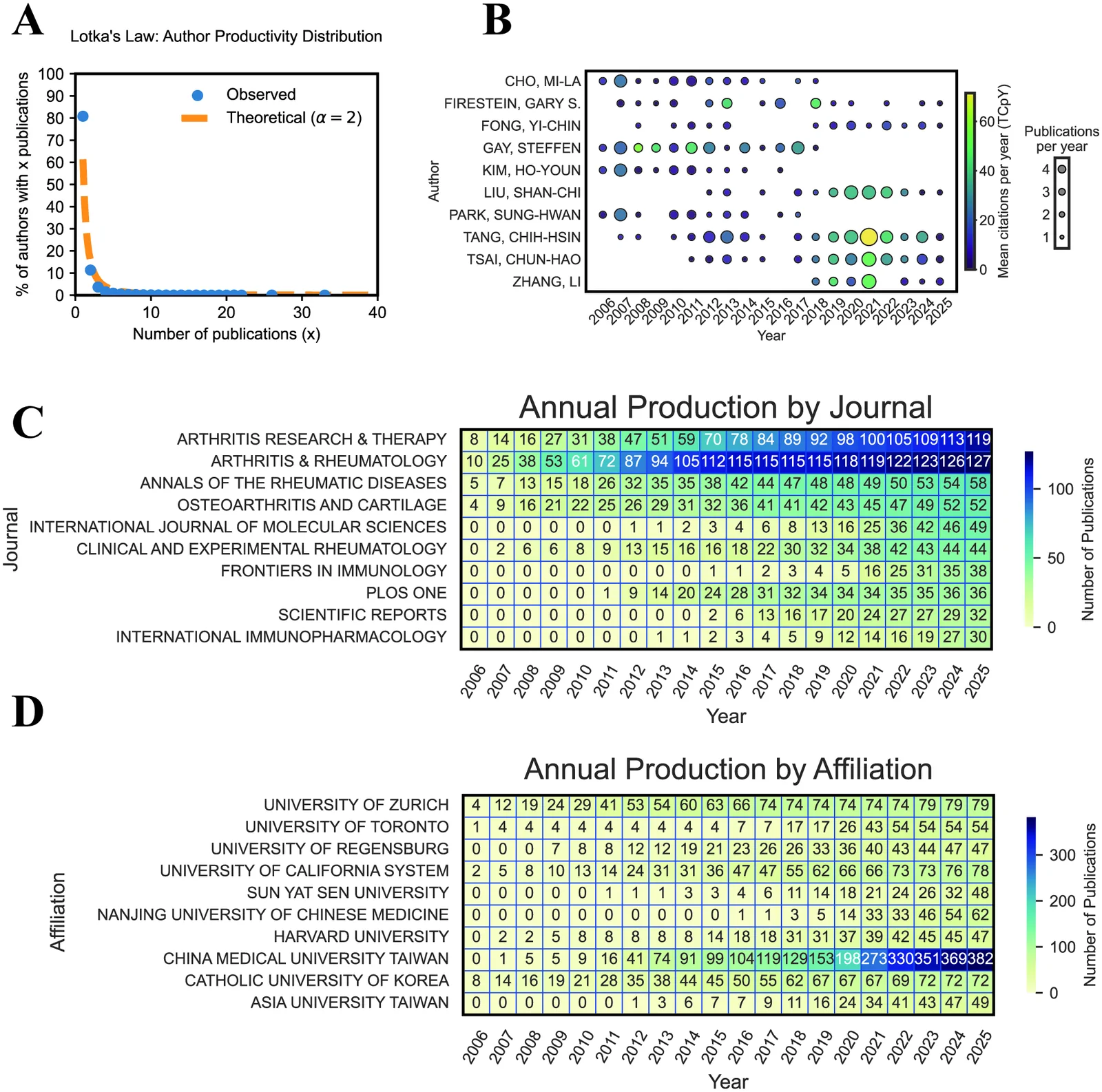
Author productivity distribution and annual output changes of journals and institutions. **(A)** Shows the distribution of author productivity fitted by Lotka’s law; **(B)** shows annual publication and citation performance of leading authors; **(C, D)** show annual publication output of leading journals and institutions, respectively.

At the author level, Tang Chih-Hsin, Gay Steffen, and Tsai Chun-Hao formed part of the core author group. Tang Chih-Hsin published 62 papers and had an h-index of 37; Gay Steffen had 4,937 total citations (TC) and an h-index of 32. Tsai Chun-Hao, Liu Shan-Chi, Cho Mi-La, Firestein Gary S., and Ospelt Caroline also showed high influence (**Table 3**; **Figure 4A**). TC indicates total citations, and NP indicates number of publications. At the same time, author productivity showed a clear long-tail distribution: 8,215 authors published only one paper, accounting for 80.8% of all authors, whereas 1,152 authors published two papers, accounting for 11.3% (**Figures 5A, B**).

**Table 3:** Prolific authors and their impact indicators.

Author	h_index	g_index	m_index	TC	NP	PY_start
TANG CHIH-HSIN	37	54	1.85	3000	62	2007
GAY STEFFEN	32	43	1.524	4937	43	2006
TSAI CHUN-HAO	27	42	1.688	1802	42	2011
LIU SHAN-CHI	24	33	1.6	1564	33	2012
CHO MI-LA	23	26	1.095	2032	26	2006
FIRESTEIN GARY S.	22	26	1.1	1987	26	2007
FONG YI-CHIN	19	21	1	959	21	2008
PARK SUNG-HWAN	19	22	0.905	1406	22	2006
OSPELT CAROLINE	18	19	0.9	1966	19	2007
KIM HO-YOUN	17	21	0.81	1338	21	2006

TC, total citations; NP, number of publications; PY_start, publication year of the first included record.

Journal distribution was relatively concentrated. Arthritis & Rheumatology, Arthritis Research & Therapy, Annals of the Rheumatic Diseases, and Osteoarthritis and Cartilage constituted the core journal group. PLOS ONE, Rheumatology, Journal of Immunology, Frontiers in Immunology, International Journal of Molecular Sciences, and International Immunopharmacology also ranked highly in publication output (**Table 4**; **Figures 4D**, **5C**).

**Table 4:** Leading journals and their impact indicators.

Source	h_index	g_index	m_index	TC	NP	PY_start
ARTHRITIS & RHEUMATOLOGY	54	90	2.571	10364	127	2006
ARTHRITIS RESEARCH & THERAPY	50	84	2.381	7545	119	2006
ANNALS OF THE RHEUMATIC DISEASES	36	58	1.714	3906	58	2006
OSTEOARTHRITIS AND CARTILAGE	30	52	1.429	2971	52	2006
PLOS ONE	24	36	1.5	1349	36	2011
RHEUMATOLOGY	23	29	1.095	1506	29	2006
JOURNAL OF IMMUNOLOGY	22	24	1.048	1969	24	2006
FRONTIERS IN IMMUNOLOGY	21	35	1.75	1237	38	2015
INTERNATIONAL JOURNAL OF MOLECULAR SCIENCES	21	44	1.4	1962	49	2012
INTERNATIONAL IMMUNOPHARMACOLOGY	18	28	1.286	807	30	2013

TC, total citations; NP, number of publications; PY_start, publication year of the first included record.

### Author and country collaboration networks

Collaboration network analysis showed that several relatively stable author collaboration clusters had formed, although links between clusters remained limited. Research teams represented by Tang Chih-Hsin, Tsai Chun-Hao, and Liu Shan-Chi, as well as teams represented by Gay Steffen, Ospelt Caroline, and Gay Renate E., were prominent in both the WOSCC and Scopus networks (**Figures 6A, B**).

**Figure 6:**
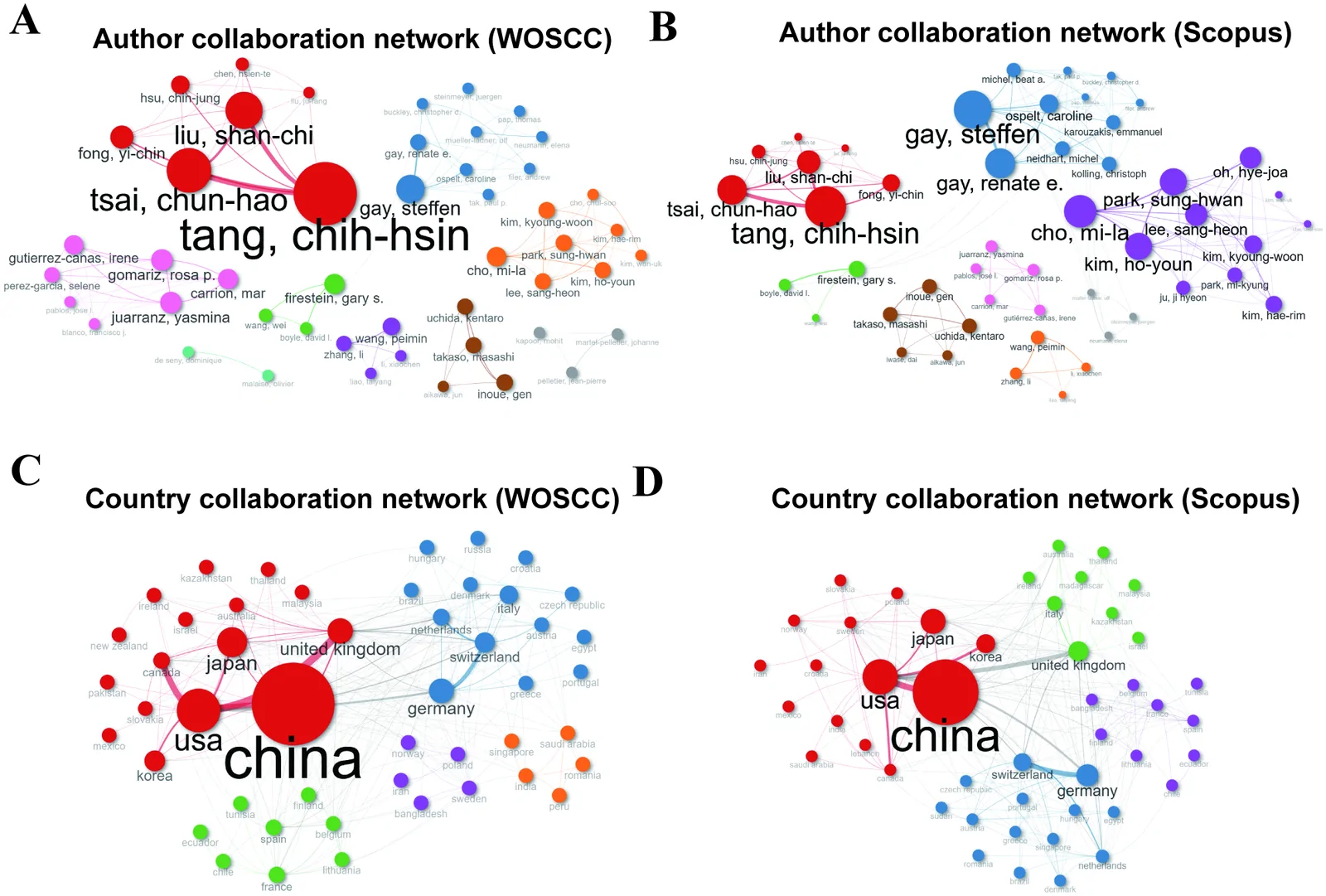
Author and country collaboration networks. **(A, B)** Show author collaboration networks in WOSCC and Scopus, respectively; **(C, D)** show country collaboration networks in WOSCC and Scopus, respectively.

In country collaboration networks, China, the United States, Japan, Germany, the United Kingdom, and Switzerland occupied important network positions, but collaboration strength and MCP% differed markedly across countries. China had the largest publication output but an MCP% of 5.6%, lower than that of the United Kingdom, Switzerland, Germany, and Italy (**Figures 4B, C**, **6C, D**; **Table 2**).

### Keyword hotspots and evolution of research frontiers

Keyword analysis showed that high-frequency terms included rheumatoid arthritis, osteoarthritis, expression, fibroblast-like synoviocytes, synovial fibroblasts, inflammation, synovium, knee osteoarthritis, NF-kappa-B, metabolism, cartilage, and apoptosis (**Figure 7B**). Rheumatoid arthritis appeared more frequently than osteoarthritis among high-frequency terms (929 vs. 561 occurrences), suggesting a strong RA-related signal in the keyword structure.

**Figure 7:**
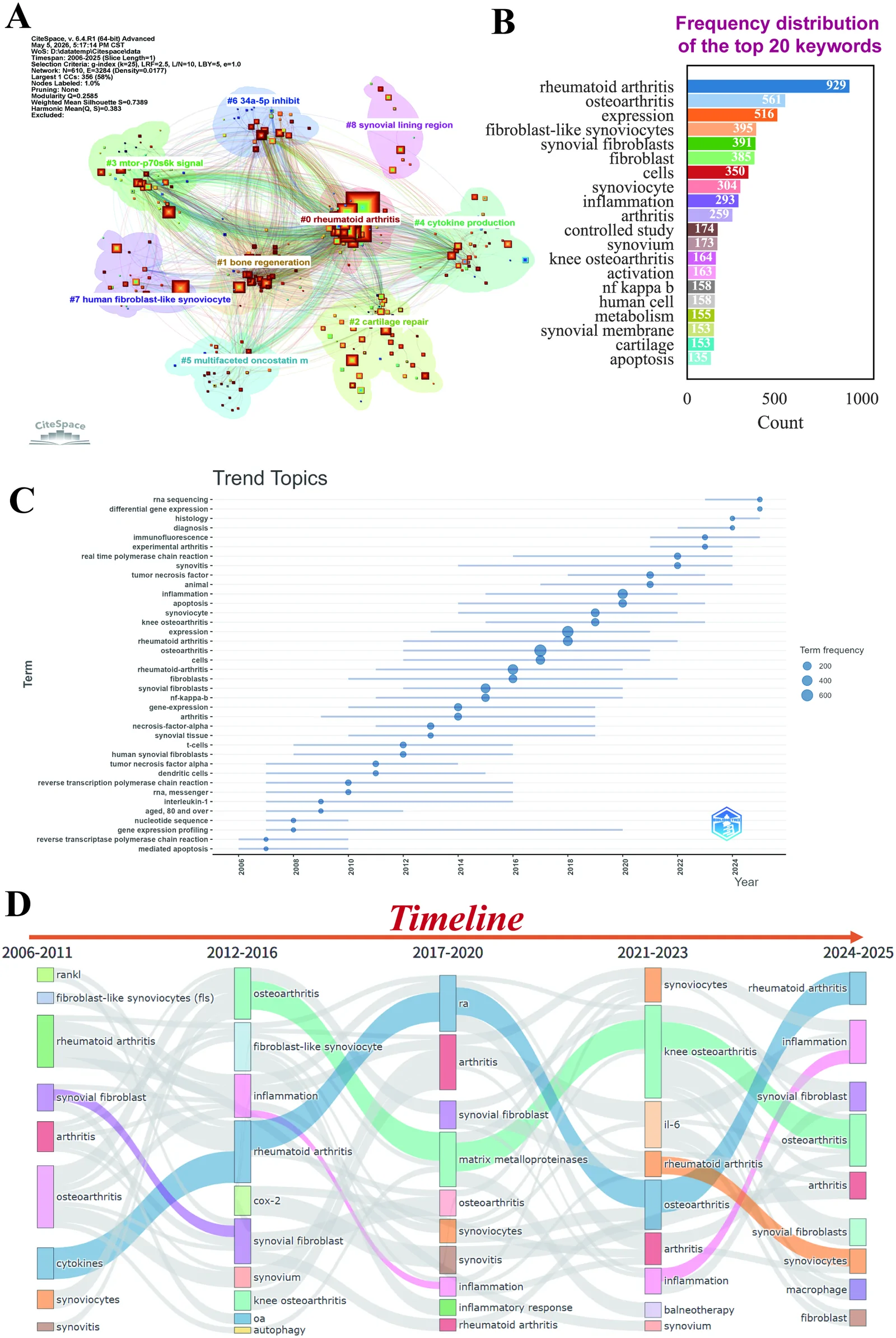
Keyword co-occurrence, high-frequency keywords, trend topics (Bibliometrix), and thematic evolution. **(A)** Shows the keyword co-occurrence network; **(B)** shows the top 20 high-frequency keywords; **(C)** shows Bibliometrix trend topics; **(D)** shows the thematic evolution timeline.

Among trend topics, early themes mainly focused on IL-1, TNF-alpha, RT-PCR, mRNA, apoptosis, and NF-kappa-B; more recent themes included synovitis, immunofluorescence, histology, diagnosis, RNA sequencing, and differential gene expression (**Figure 7C**). In the keyword clustering and thematic evolution maps, terms such as macrophage, fibroblast, synovial lining region, metabolism, and autophagy appeared persistently (**Figures 7A, D**). Overall, research hotspots have expanded from inflammatory mediators and pathway detection to synovial tissue structure, cellular heterogeneity, immune-stromal interactions, and tissue microenvironment-related topics.

### Citation dynamics and knowledge structure

Globally highly cited documents and highly cited documents within the included corpus were not fully overlapping. The documents with the highest total citations included Khandpur 2013, Boilard 2010, Stanczyk 2008, Nakasa 2008, and Gupta 2019, which covered broader topics such as NETs, platelet microparticles, microRNA, inflammatory arthritis, and cross-species joint disease reviews (**Figure 8A**; **Table 5**).

**Figure 8:**
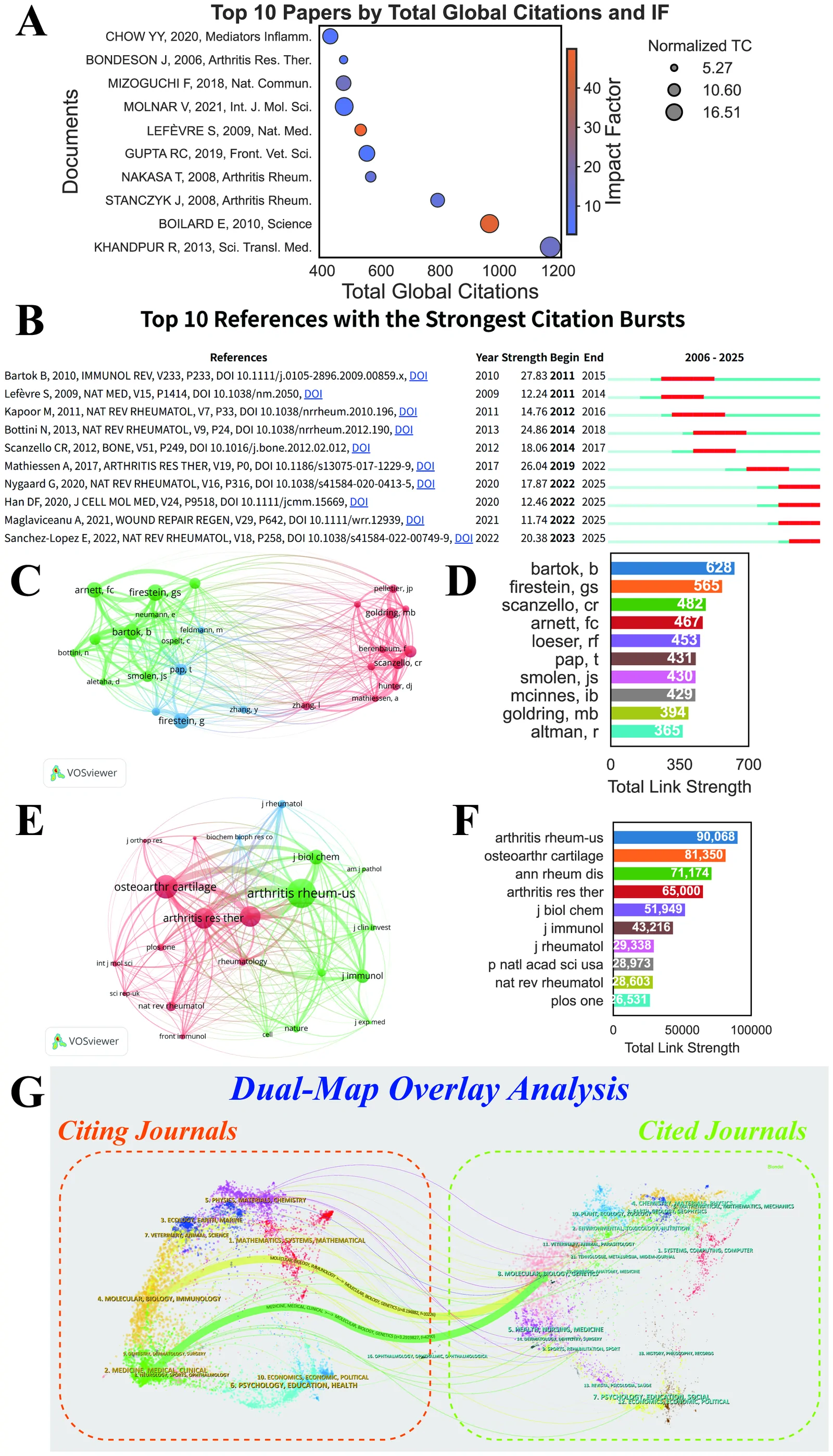
Citation structure, burst references, co-citation networks, and disciplinary knowledge flow. **(A)** Shows documents with the highest total citations; **(B)** shows references with the strongest citation bursts; **(C–F)** show author and journal co-citation networks and total link strength; **(G)** shows citation paths in the dual-map overlay analysis.

**Table 5:** Globally highly cited documents.

Paper	DOI	Total citations	TC per year	Normalized TC	IF
KHANDPUR R, 2013, SCI TRANSL MED	10.1126/scitranslmed.3005580	1173	83.79	16.51	14.6
BOILARD E, 2010, SCIENCE	10.1126/science.1181928	967	56.88	14.19	45.7
STANCZYK J, 2008, ARTHRITIS RHEUM	10.1002/art.23386	791	41.63	9.46	10.8
NAKASA T, 2008, ARTHRITIS RHEUM-US	10.1002/art.23429	564	29.68	6.75	10.8
GUPTA RC, 2019, FRONT VET SCI	10.3389/fvets.2019.00192	551	68.88	11.43	2.8
LEFÈVRE S, 2009, NAT MED	10.1038/nm.2050	530	29.44	7.58	49.9
MOLNAR V, 2021, INT J MOL SCI	10.3390/ijms22179208	474	79	13.81	4.9
MIZOGUCHI F, 2018, NAT COMMUN	10.1038/s41467-018-02892-y	472	52.44	10.33	15.7
BONDESON J, 2006, ARTHRITIS RES THER	10.1186/ar2099	472	22.48	5.27	4.6
CHOW YY, 2020, MEDIAT INFLAMM	10.1155/2020/8293921	427	61	10.87	4.2

By contrast, documents ranked by Local Citations were more concentrated around the internal knowledge core of the OA-FLS dataset. Lefèvre 2009 had the highest number of Local Citations; Bondeson 2006, Ospelt 2008, Stanczyk 2008, Han 2020, Mizoguchi 2018, Maglaviceanu 2021, Wang 2006, Kato 2014, Remst 2014, and Nanus 2021 also ranked highly by this metric (**Table 6**). These locally highly cited documents involved RA-FLS migration and invasion, TLR- and microRNA-related RA-FLS frameworks, OA synovitis, early OA-FLS responses, exosome-mediated cartilage crosstalk, synovial fibrosis, FLS-related synovitis, and pain-associated fibroblast subsets.

**Table 6:** Highly cited documents ranked by local citations.

Paper	DOI	Year	Local citations	Global citations	LC/GC ratio (%)	Normalized local citations	Normalized global citations
LEFÈVRE S, 2009, NAT MED	10.1038/nm.2050	2009	77	530	14.53	18.28	7.58
BONDESON J, 2006, ARTHRITIS RES THER	10.1186/ar2099	2006	39	472	8.26	8.87	5.27
OSPELT C, 2008, ARTHRITIS RHEUM-US	10.1002/art.24140	2008	38	295	12.88	9.73	3.53
STANCZYK J, 2008, ARTHRITIS RHEUM	10.1002/art.23386	2008	36	791	4.55	9.22	9.46
HAN DF, 2020, J CELL MOL MED	10.1111/jcmm.15669	2020	36	117	30.77	13.25	2.98
MIZOGUCHI F, 2018, NAT COMMUN	10.1038/s41467-018-02892-y	2018	35	472	7.42	14	10.33
MAGLAVICEANU A, 2021, WOUND REPAIR REGEN	10.1111/wrr.12939	2021	34	91	37.36	16.81	2.65
WANG CT, 2006, OSTEOARTHR CARTILAGE	10.1016/j.joca.2006.05.009	2006	32	220	14.55	7.28	2.46
TANG CH, 2007, J IMMUNOL	10.4049/jimmunol.179.8.5483	2007	32	233	13.73	9.23	3.99
KATO T, 2014, ARTHRITIS RES THER	10.1186/ar4679	2014	28	272	10.29	9.27	5.34
REMST DFG, 2014, ARTHRITIS RHEUMATOL	10.1002/art.38266	2014	28	112	25	9.27	2.2
STANCZYK J, 2011, ARTHRITIS RHEUM-US	10.1002/art.30115	2011	27	296	9.12	9.38	5.05
KAROUZAKIS E, 2009, ARTHRITIS RHEUM-US	10.1002/art.25018	2009	26	266	9.77	6.17	3.8
NAKANO K, 2013, ANN RHEUM DIS	10.1136/annrheumdis-2012-201526	2013	24	312	7.69	7.66	4.39
TANG CH, 2010, ARTHRITIS RHEUM-US	10.1002/art.27755	2010	23	90	25.56	9.93	1.32
KUO SJ, 2017, PLOS ONE	10.1371/journal.pone.0176052	2017	23	35	65.71	8.68	0.77
ZHANG L, 2019, OXID MED CELL LONGEV	10.1155/2019/6326517	2019	23	119	19.33	7.36	2.47
GARCIA-CARBONELL R, 2016, ARTHRITIS RHEUMATOL	10.1002/art.39608	2016	22	279	7.89	9.13	6.21
STEENVOORDEN MMC, 2006, ARTHRITIS RES THER	10.1186/ar2073	2006	21	84	25	4.78	0.94
NANUS DE, 2021, EBIOMEDICINE	10.1016/j.ebiom.2021.103618	2021	21	116	18.1	10.38	3.38

Co-citation networks further defined the knowledge base of the field. Bartok, Firestein, Scanzello, Loeser, Pap, Smolen, McInnes, Goldring, and Altman showed high link strength; co-cited journals were concentrated in Arthritis Rheum-US, Osteoarthritis and Cartilage, Annals of the Rheumatic Diseases, Arthritis Research & Therapy, Journal of Immunology, and Journal of Biological Chemistry (**Figures 8C–F**).

### Disciplinary knowledge flow and thematic evolution

Dual-map overlay analysis showed that OA-FLS research connected knowledge domains including medicine/clinical science, molecular biology/immunology, health care, and molecular biology/genetics. Citation paths were mainly distributed between clinical medicine and molecular biology, immunology, and genetics (**Figure 8G**). In other words, the main paths from citing journals on the left to cited journals on the right indicated that knowledge integration in this field is mainly driven from clinical joint disease questions toward molecular, immunological, and genetic explanations.

Integrating keywords, trend topics, and citation structures, the thematic evolution of OA-FLS research can be summarized into three periods. Representative themes from 2006 to 2012 included PCR, mRNA, IL-1, TNF-alpha, and NF-kappa-B; from 2013 to 2020, terms such as MMPs, COX-2, IL-6, autophagy, metabolism, and knee osteoarthritis increased; from 2021 to 2025, themes such as RNA sequencing, differential gene expression, histology, immunofluorescence, macrophage, and fibroblast appeared persistently (**Figure 7**; citation burst results are shown in **Figure 8B**).

## Discussion

### Principal findings and field position

Based on integrated WOSCC and Scopus data, this study systematically mapped the knowledge structure and thematic evolution of OA-FLS research from 2006 to 2025. The field showed steady publication growth, identifiable core authors, institutions, and journals, but limited international collaboration and cross-team validation. Keywords, trend topics, and locally highly cited documents also indicate a change in research emphasis. Early work mainly focused on molecular responses under classical inflammatory stimulation. Recent studies increasingly address FLS states, cellular interactions, and symptom-related questions in patient tissues. This thematic shift is more informative than publication growth alone.

This shift is internally consistent with the broader context of OA research. OA has gradually been reframed from a “cartilage degeneration disease” to a whole-joint organ disease, and FLS are located at the intersection of synovitis, cartilage injury, matrix remodeling, and the pain microenvironment. Therefore, the value of OA-FLS research should not stop at demonstrating that these cells can express certain inflammatory molecules. Rather, it lies in explaining how they participate in OA heterogeneity within the real local synovial environment (27, 28). These patterns help explain why OA-FLS research is moving beyond single-pathway studies and toward questions grounded in patient tissue.

The following interpretation is therefore anchored in keyword frequency, trend-topic distribution, local citation structure, co-citation patterns, and collaboration indicators, rather than in *de novo* experimental evidence.

### From inflammatory response to disease-contextual interpretation

From the literature structure, the early focus of OA-FLS research was clear: to determine whether OA-FLS could play an active pathological role in OA. Early high-frequency themes such as IL-1, TNF-alpha, NF-kappa-B, MMPs, and mRNA expression aligned with studies that entered the locally highly cited core around 2006. These studies largely used *in vitro* models to assess OA-FLS responses to inflammatory or metabolic stimuli (29, 30). Bibliometrically, this cluster marks an early emphasis on inflammatory and catabolic responses.

However, *in vitro* stimulation models have clear boundaries. They can answer whether cells have inflammatory responsiveness, but they are less able to explain why different patients show varying degrees of synovitis, pain, and structural progression. The early inflammatory-catabolic framework was therefore important, but it could not fully account for the cellular states and tissue ecology observed in patient synovium.

Recent thematic changes reflect this shift. The rise of synovitis, histology, immunofluorescence, RNA sequencing, differential gene expression, macrophage, and fibroblast suggests a move beyond asking what cells express. Attention now includes cellular location, interactions, and links to disease phenotypes. Thus, early inflammatory pathways have not been replaced; rather, they are being reinterpreted within tissue location, cellular states, and clinical phenotypes (27, 28).

### RA-FLS knowledge source and OA-contextualized interpretation

The high frequency of rheumatoid arthritis and the prominent position of several classic RA-FLS studies in the locally highly cited network indicate that OA-FLS research has long been shaped by the RA research framework. This pattern should not be viewed simply as “noise” or database bias; it is better understood as a historical trace of disciplinary development. RA-FLS research characterized these cells as active pathological participants earlier and provided a relatively mature analytical language for later studies, including cellular activation, invasion-like phenotypes, TLR/NF-kappa-B signaling, epigenetic regulation, and fibroblast subsets (31–33).

For OA research, which has long been dominated by a cartilage-centered model, these conceptual resources are informative. The issue is that the core pathology of RA is persistent autoimmune inflammation, whereas OA more often reflects the convergence of mechanical loading, aging, metabolic stress, cartilage injury, and chronic tissue remodeling. Therefore, the same signaling pathway or cellular state term does not necessarily have the same tissue origin, mode of persistence, or clinical meaning in OA as in RA. RA-FLS is more appropriately viewed as a conceptual source for OA-FLS rather than as a disease-equivalent model (34).

These findings do not prove a linear replacement of RA-derived concepts by OA-specific frameworks. High-frequency RA-related terms and RA-FLS papers in the local citation core indicate continued conceptual borrowing. At the same time, recent OA-oriented topics such as synovitis, fibrosis, macrophage-FLS interaction, pain-associated fibroblast subsets, RNA sequencing, and histology suggest increasingly OA-contextualized interpretation. Future studies should therefore test where RA-derived concepts apply in OA tissues and where they need to be redefined, rather than assuming that the same pathway or cell-state term has the same meaning in both diseases.

### Intracellular regulation: non-coding RNA, epigenetics, and cellular programs

Global and local citation structures place microRNA, other non-coding RNAs, and epigenetic studies in a stable position within OA-FLS research. The prominence of Stanczyk and related studies reflects a shift from post-stimulation expression changes toward post-transcriptional regulation (11). Related work links this shift to the maintenance of FLS states (32, 35, 36). Bibliometrically, this theme is relevant to the persistence of altered FLS programs in OA.

Keyword and citation structures suggest that the value of this work lies in explaining how FLS states may be stabilized, rather than in adding isolated RNA-target relationships. DNA methylation and methylome studies may contribute to the maintenance of abnormal FLS phenotypes (35, 36). OA synovial miRNA studies also link non-coding RNA regulation to inflammation, matrix deposition, and cell proliferation (37). This theme is better interpreted as a transition from pathway response to cell-state programs in OA-FLS research.

At present, however, this direction remains fragmented, often moving from one RNA to one target and several functional indicators. These findings will be easier to compare across studies if they are linked to defined inflammatory, matrix-remodeling, fibrotic, or pain-associated FLS states.

### Cartilage-synovium crosstalk, extracellular vesicles, and matrix remodeling

Taken together, the local citation structure and recurrent keyword themes indicate that OA-FLS research is moving beyond intracellular responses and increasingly repositioning FLS within the tissue-pathological setting of OA.

Within the OA joint, FLS and chondrocytes are linked bidirectionally. Cartilage-derived matrix fragments, damage-associated signals, and metabolic stress can activate synovial cells. Conversely, OA-FLS may influence chondrocyte catabolism through inflammatory mediators, prostaglandins, matrix-degrading enzymes, and chemokines. This supports a view of the synovium as an active participant in local inflammation and matrix degradation (38, 39).

Extracellular vesicle research reinforces this bidirectional view. In the local citation and keyword structures, vesicle-mediated effects on inflammatory responses, apoptosis, and matrix metabolism position intercellular communication as an emerging cartilage-synovium theme (40–42).

Synovial fibrosis and matrix remodeling also recur as OA-FLS themes (43). Studies associate these phenotypes with TGF-beta, mechanical stress, hypoxia, and altered collagen, MMP, TIMP, and ECM programs (43, 44). Their persistence in the literature suggests a chronic remodeling axis rather than a transient inflammatory effect.

### Immune-stromal niches and pain-associated fibroblast states

The convergence of macrophage and fibroblast in the keyword network, together with the locally highly cited Nanus study, suggests a move toward local niche interpretation. Within this bibliometric pattern, OA synovitis is increasingly discussed as an inflammatory-stromal process involving multiple cell and matrix components.

Within this niche, macrophages and FLS may amplify each other. Macrophage-derived mediators can induce chemokine and matrix-remodeling responses in FLS (29). FLS-derived vesicles and chemotactic signals may, in turn, influence macrophage activation and recruitment (42). This reciprocal pattern is consistent with the convergence of macrophage and fibroblast terms in the keyword network.

Together, the keyword and local citation patterns link inflammation and tissue remodeling with symptom-related OA-FLS research.

Pain-associated FLS are clinically relevant because OA pain is often incompletely aligned with imaging-defined structural damage (45). The locally highly cited Nanus study identified distinct fibroblast subsets and inflammatory or pain-related features at painful synovial sites (9). These features may contribute to nociceptor sensitization (9, 46). Related fibrotic and matrix-remodeling states may also affect neural-stromal interactions (9, 47). Its position in the local citation structure makes pain-associated FLS an internal OA-FLS theme rather than a purely clinical adjunct.

This pattern links FLS biology more directly to patient-relevant symptoms.

### Single-cell and spatial technologies: opportunities and current gaps

RNA sequencing, differential gene expression, histology, and immunofluorescence have increased in recent years. This trend reflects a move from average expression differences toward higher-resolution cellular and tissue evidence. Single-cell sequencing reveals heterogeneity, while spatial omics and multiplex immunofluorescence add localization (27, 28, 48).

However, technical vocabulary does not mean mechanisms are resolved. FLS subset nomenclature, sampling site, processing workflow, sequencing platform, batch effects, and incomplete clinical phenotyping still limit comparability across studies (49–51). Thus, high-resolution technologies provide new observational windows, but the biological and clinical meaning of OA-FLS states still requires validation.

### Collaboration, standardization, and validation

China contributed the largest number of publications, but its MCP% was markedly lower than that of the United Kingdom and Switzerland. This collaboration indicator suggests a need for more substantive international work on FLS state definitions, tissue-processing workflows, and clinical phenotype anchoring. Such collaboration becomes especially important as the field moves toward cross-cohort and cross-platform replication. Thus, low MCP% suggests room for stronger multicenter validation and standard setting in high-output countries.

### Future perspectives

The next step is to make existing mechanistic signals more reproducible and clinically interpretable. The central task is to determine which FLS states have stable pathological meaning in defined tissue locations and clinical phenotypes.

First, a clearer minimum reporting framework and multicenter validation system should be developed. OA-FLS definitions are affected by joint site, disease stage, sampling region, tissue digestion, culture passage, sequencing platform, and annotation standards. Future studies should report these variables consistently. Key metadata include sample source, OA stage, imaging-defined synovitis, histological scores, pain phenotype, medication background, cell isolation, culture information, and spatial validation.

Second, paired-tissue and phenotype-driven designs are needed. OA-FLS should be studied within the whole-joint disease unit rather than only *in vitro*. Ideally, studies should combine synovium, cartilage, synovial fluid, imaging, histology, and symptom information from the same patient.

Third, macrophage-FLS interactions, cartilage-synovium crosstalk, fibrotic remodeling, and pain-associated FLS should be treated as verifiable research units. Their pathological meaning requires stable identification and spatial support in patient tissues. Cross-validation against pain, imaging-defined synovitis, or progression outcomes is also needed.

Finally, single-cell discoveries should follow a continuous evidence chain. This includes state identification, spatial localization, integration with synovial fluid, cartilage, imaging, and clinical data, and functional validation through co-culture, animal models, human tissue models, or intervention experiments. Emerging three-dimensional synovial organoids, joint organoids, organ-on-chip platforms, and related non-animal human tissue models may help bridge descriptive multi-omics and functional validation. When informed by single-cell and spatial multi-omics, these models may provide more direct platforms for testing disease-associated OA-FLS states, immune-stromal interactions, biomarker candidates, and therapeutic responses (52, 53). Such a sequence would make single-cell findings more useful than cluster naming alone.

Future bibliometric analyses may also need to reflect the evolving nomenclature of OA-FLS heterogeneity. As single-cell and spatial studies refine fibroblast-state definitions, publications could be stratified by emerging location or state labels, such as lining versus sublining fibroblasts and inflammatory versus matrix-remodeling states, allowing future science-mapping studies to follow how OA stromal concepts mature over time.

In practical terms, the pathological significance of an OA-FLS state should not be inferred from marker expression alone. It should be supported by four layers of evidence: recurrence across independent cohorts, localization in relevant synovial tissue regions, functional evidence from perturbation or co-culture models, and association with clinical phenotypes. Relevant phenotypes may include synovitis, pain, structural progression, or treatment response. An FLS state becomes useful for explaining OA heterogeneity only when it can be localized in tissue, tested functionally, and linked consistently to patient phenotypes.

### Limitations

This study has several limitations. First, bibliometric analysis is inevitably affected by database coverage, search strategy, and metadata quality. Although this study integrated WOSCC and Scopus, it may still have missed non-English publications, recent studies not yet fully indexed, preprints, or materials outside these databases. Therefore, database coverage bias and language bias cannot be fully excluded.

Second, to ensure thematic specificity, the search strategy was mainly constructed around synovial fibroblasts and FLS. It may therefore not have fully captured studies that used terms such as stromal cells, mesenchymal stromal cells, synovial lining cells, or other stromal-cell terms but substantively involved synovial fibroblast states. This decision reduced terminological heterogeneity in the retrieved corpus but may have led to underrepresentation of studies indexed under broader stromal-cell terminology.

Third, the dataset may overrepresent RA-FLS-related literature because RA-FLS research has historically provided many of the conceptual and methodological references for synovial fibroblast studies. Therefore, the strong RA signal observed in keyword and citation structures should be interpreted both as a historical knowledge source and as a potential source of conceptual bias.

Fourth, keyword, citation, and co-citation networks can reflect research attention and knowledge structure, but they cannot replace experimental evidence. The discussion of shifts in OA-FLS research in this article should therefore be understood as a cautious inference based on literature structure rather than as direct proof of any mechanism. Fifth, citations are time-lagged; earlier publications are more likely to enter the highly cited core, whereas the true influence of recent studies requires time to emerge. Finally, terms such as OA-FLS, synovial fibroblasts, FLS, synoviocytes, and stromal cells are not used consistently across studies. At the same time, the rise of terms related to single-cell analysis, spatial omics, histology, and immunofluorescence reflects both changes in research questions and the diffusion of technologies themselves. Interpretation of these trends still requires paired tissue samples, functional studies, and cross-cohort replication.

## Conclusion

This dual-database bibliometric analysis characterized the knowledge structure of OA-FLS research from 2006 to 2025. The field has expanded from inflammatory activation and catabolic-response studies toward FLS functional states, immune-stromal interactions, cartilage-synovium crosstalk, matrix remodeling, and symptom-related questions. RA-FLS-related literature remains an important knowledge source, but recent keyword, trend-topic, and local-citation patterns suggest increasing attention to OA-contextualized tissue remodeling, synovial niches, and clinical heterogeneity. These findings should be interpreted as bibliometric evidence of thematic movement rather than direct proof of mechanistic transition.

## Data Availability

The original contributions presented in the study are included in the article/**Supplementary Material**. Further inquiries can be directed to the corresponding authors.

## Supplementary material

Supplementary Table 1 Provides the database search strategies and retrieval details used for the bibliometric analysis.
