# Supplementary material for: Synovial inflammation and immune-stromal crosstalk in osteoarthritis-related synovial fibroblast research, 2006–2025: a bibliometric analysis
Source: Front Immunol. 2026 Oct 1;17:1898740. doi: 10.3389/fimmu.2026.1898740 (PMC13669094; doi:10.3389/fimmu.2026.1898740)
Supplement: Supplementary file 1 [file Table1.docx]

***Supplementary Material***

Supplementary Table S1 | WOSCC and Scopus search strategies, database restrictions, and retrieval details.

| **Item** | **Details** |
| --- | --- |
| **WOSCC database** | Web of Science Core Collection |
| **WOSCC field** | Topic Search |
| **WOSCC search strategy** | TS=(("osteoarthrit*" OR "osteoarthros*" OR "degenerative joint disease*" OR "degenerative arthritis") AND ("synovial fibroblast*" OR "fibroblast-like synoviocyte*" OR "fibroblast like synoviocyte*" OR "fibroblast-like synovial cell*" OR "fibroblast like synovial cell*" OR "type B synoviocyte*" OR "type-B synoviocyte*" OR "osteoarthritis synovial fibroblast*" OR "osteoarthritic synovial fibroblast*" OR "osteoarthritis fibroblast-like synoviocyte*" OR "osteoarthritic fibroblast-like synoviocyte*")) |
| **Scopus database** | Scopus |
| **Scopus field** | Title/Abstract/Keywords |
| **Scopus search strategy** | TITLE-ABS-KEY(("osteoarthrit*" OR "osteoarthros*" OR "degenerative joint disease*" OR "degenerative arthritis")) AND TITLE-ABS-KEY(("synovial fibroblast*" OR "fibroblast-like synoviocyte*" OR "fibroblast like synoviocyte*" OR "fibroblast-like synovial cell*" OR "fibroblast like synovial cell*" OR "type B synoviocyte*" OR "type-B synoviocyte*" OR "osteoarthritis synovial fibroblast*" OR "osteoarthritic synovial fibroblast*" OR "osteoarthritis fibroblast-like synoviocyte*" OR "osteoarthritic fibroblast-like synoviocyte*")) |
| **Database restrictions** | Publication years were restricted to January 1, 2006, through December 31, 2025. |
| **Search date** | All searches and data exports were completed on May 2, 2026. |
| **Document type and language filters** | English-language Articles and Review Articles. |
| **Initial records** | WOSCC: 1,592 records; Scopus: 1,339 records. |
| **Deduplication and final corpus** | A total of 1,050 duplicated publications were removed; 1,881 unique publications were included, comprising 1,642 articles and 239 reviews. |
